# Golden Barrel Cactus: Unveiling Its Potential as a Functional Food and Nutraceutical Source

**DOI:** 10.3390/foods13071137

**Published:** 2024-04-08

**Authors:** Nipha Chaicharoenaudomrung, Kakanang Posridee, Anant Oonsivilai, Ratchadaporn Oonsivilai

**Affiliations:** 1 School of Food Technology, Institute of Agricultural Technology, Suranaree University of Technology, Nakhon Ratchasima 30000, Thailand; nipha.chai@gmail.com (N.C.); posridee.ka@gmail.com (K.P.); 2 School of Electrical Engineering, Institute of Engineering, Suranaree University of Technology, Nakhon Ratchasima 30000, Thailand; 3 Health and Wellness Research Unit, Suranaree University of Technology, Nakhon Ratchasima 30000, Thailand

**Keywords:** golden barrel cactus, phytochemical, digestive stability, bioaccessibility, phenolic

## Abstract

A comprehensive analysis of phytochemicals, digestive stability, and bioaccessibility was conducted on a golden barrel cactus extract from 3- and 6-year-old plants. Both ages contained lutein and four chlorophyll derivatives (chlorophyll *a*, *b*, pheophytin *a*, and *b*), but younger cacti revealed a significantly higher abundance. Total phenolics reached 3545.35 mg gallic acid equivalent/100 g dry weight in the 3-year-old extracts compared to 2557.96 mg/100 g in the older ones. Antioxidant activity, assessed by DPPH, ABTS, and FRAP assays, was consistently higher in the younger group. While digestion impacted all compounds, lutein exhibited relative stability at 69.03% and 58.33% retention for 3- and 6-year-old extracts, respectively. Chlorophylls displayed lower stability (37.64% and 33.34% remaining for younger and older cacti) despite showing higher bioaccessibility (73.385% and 64.65%). Phenolics also demonstrated promising bioaccessibility (76.39% and 69.88%) despite moderate digestive degradation (60.52% and 56.89% retained). Conclusively, all investigated attributes—phytochemical content, digestive stability, and bioaccessibility—favored the younger golden barrel cactus extracts. This highlights the crucial role of plant age in maximizing the potential health benefits of these extracts.

## 1. Introduction

The Golden Barrel Cactus (*Echinocactus grusonii*, GBC), native to Mexico, has garnered attention for its rich phytochemical profile, boasting lutein, chlorophylls, phenolic acids, flavonoids, and betalains [1]. These bioactive compounds contribute to its impressive antioxidant activity, evidenced by potent free radical scavenging capacity in DPPH and ABTS assays, surpassing even established antioxidant sources like berries [2,3]. However, challenges lie in bioavailability, with in vitro studies revealing moderate cellular uptake of chlorophylls and phenolics from GBC extracts in Caco-2 cells, while lutein exhibited better absorption potential [4]. Despite these limitations, GBC holds promise as a novel functional food ingredient and nutraceutical source, warranting further exploration of its health benefits and optimal utilization strategies.

De Leo et al. [5] explored the phytochemical profile of this Mexican native plant, now cultivated in several semiarid regions, highlighting its fruit’s richness in ascorbic acid and polyphenols. Research on cactus extract, particularly from *Opuntia ficus-indica*, has revealed a wealth of beneficial phytochemicals with applications in health and medicine [6]. Furthermore, Galati et al. [6] demonstrated that *O. ficus-indica* gel can coat the stomach lining, protecting against alcohol-induced peptic ulcers in animal models. Studies like Hernández-Urbiola et al.’s [7] analysis of Nopal (*O. ficus-indica*) at various maturity stages further solidify its value as a source of calcium and dietary fiber, particularly at later stages (135 days). This suggests a potential for Nopal as an economical food supplement with the ability to ameliorate or prevent chronic and degenerative diseases. Notably, NeOpuntia^®^, a trademarked blend of soluble and insoluble fibers from *O. ficus-indica* leaves, has been shown to possess hypolipemic properties, beneficial for individuals with lipid metabolism disorders. Furthermore, research utilizing in vitro digestion models has been conducted to assess the gastrointestinal stability and bioavailability of cactus-derived nutrients, phytochemicals, and phenolic compounds. [8,9,10,11]. This approach holds promise for assessing the bioavailability of nutrients and phytochemicals in various foods.

Turning our attention to the Golden Barrel Cactus (*Echinocactus grusonii*), commonly known as “Tang Thong” in Thailand, further research opportunities emerge. Like *O. ficus-indica*, this Mexican native cactus belonging to the Cactaceae family has gained immense popularity in Thailand. However, information regarding its phytochemical profile and bioactivities remains relatively limited.

By drawing parallels between *O. ficus-indica* and Tang Thong, and building upon existing research on cactus extract, further exploration into the bioactive compounds and potential health benefits of this very interesting GBC is warranted.

## 2. Materials and Methods

### 2.1. Materials

Cacti specimens of GBC aged 3 and 6 years were collected from Uncle Chorn’s Cabin Garden, Pathumthani province, Thailand. The plant material was subsequently subjected to a rigorous preparation protocol, encompassing trimming, cleaning, chopping, and tray drying at 60 °C (Kluay Nam Thai Tow Op, Bangkok, Thailand). Following dehydration, the cacti were mechanically ground using an Ultra Centrifugal Mill Model ZM-1000 (Retsch, Haan, Germany) and sieved through a 0.2 mm mesh to obtain a homogenous powder. The prepared cactus powder was then vacuum-packaged and stored at −20 °C for subsequent utilization in the research study.

### 2.2. Preparation of Acetonitrile Extract of GBC

The GBC powder was extracted using a modification of the method described by [12]. Briefly, 500 mg of the powder was dissolved in 15 mL of acetonitrile and subjected to an ultrasonic bath (Elma Ultrasonic, Singen, Germany) treatment for 20 min at 25 °C. Subsequently, the mixture was centrifuged at 3000× *g* for 10 min (Thermo Electron LED GmbH, Langenselbold, Germany) and filtered through Whatman no. 1 paper. To obtain a higher yield of the desired compounds, the cactus material was extracted twice using the same process. The extracted liquids from both rounds were then combined, and the final volume was adjusted to 50 mL. A rotary evaporator (Buchi Rotavapor R-114, Santa Clara, CA, USA) was used to gently remove the solvent from the extract.

### 2.3. Phytochemical Profile of GBC Crude Extract

#### 2.3.1. Chlorophyll and Lutein Analysis

Following a modified protocol by [12], *Echinocactus grusonii* powder (500 mg) was extracted twice with acetonitrile via sonication (25 °C, 20 min) and centrifugation (3000× *g*, 10 min). Supernatants were combined, filtered (Whatman No. 1), and concentrated by rotary evaporation.

#### 2.3.2. Total Phenolic Compounds Quantification

Following the modified Folin-Ciocalteu technique of Oonsivilai et al. [13], the total phenolic content of the samples was determined. They were quantified using duplicate 0.02 mL aliquots of gallic acid standard, sample, or blanks, and they reacted with Folin-Ciocalteu/Na_2_CO_3_. After a 2 h incubation in the dark, absorbance was measured at 765 nm (Libra S22, Thermo Fisher Scientific Inc., Bangkok, Thailand). A 0–750 mg/L gallic acid standard curve facilitated quantification, expressed as mg GAE/100 g DW.

### 2.4. Fiber Antioxidant Activities

#### 2.4.1. DPPH Free Radical Scavenging Activity

DPPH radical scavenging activity was determined according to [14] by mixing 0.1 mL of standard/sample/blank with 1.90 mL DPPH solution in test tubes. Scavenging activity was quantified by absorbance measurement at a wavelength of 515 nm.

#### 2.4.2. Scavenging Activity of ABTS Radical Cation

This study employed the ABTS radical scavenging assay reported by [15] to assess the antioxidant potential of the extract. Reaction mixtures containing 1900 μL of ABTS^+^ solution, 100 μL of serially diluted extract, and distilled water (control) were incubated for 6 min after homogenization. Their absorbance was then measured at 734 nm, along with similar mixtures containing BHT and ascorbic acid as standard controls. The extracts’ ABTS scavenging ability was expressed as IC_50_, which was the concentration at which they inhibited 50% of the ABTS^+^ radicals.

#### 2.4.3. Ferric Reducing Antioxidant Power Assay

Following the modified FRAP protocol based on [16,17], total antioxidant capacity was quantified. The sample (50 µL) and water (150 µL) were added to 1.5 mL of the FRAP reagent. After 4 min, absorbance at 593 nm was recorded. A ferrous sulfate standard curve (100–1000 µM) enabled the expression of results as mM ferrous equivalents/g of dry weight (in triplicate measurements).

### 2.5. Bioaccessibility and Bioavailability

#### 2.5.1. In Vitro Digestion

This study employed a modified version of the simulated digestion protocols established by [18,19] to analyze the GBC crude extract.

##### Gastric Phase

In a 50 mL polypropylene tube, 2 g of dried GBC powder was suspended in 20 mL of a 120 mM NaCl solution containing 5% (*v*/*v*) Tween-80. This mixture was homogenized using a model T25D homogenizer (Laboratory Supply Network, Inc., Atkinson, Germany) to ensure uniformity. Subsequently, the pH was adjusted to 2.0 ± 0.1 using 1 M HCl solution to mimic the acidic environment of the stomach. Peptide hydrolysis was initiated by the addition of 2 mL of porcine pepsin solution (40 mg/mL, 100 mM HCl), followed by volume adjustment to 40 mL with a 120 mM NaCl solution. To minimize external contamination, the tube was purged with nitrogen gas and tightly capped using Parafilm before incubation in a water bath (JULABO SW22, Marshall Scientific, Cambridge, MA, USA) at 37 °C for 1 h at 95 rpm.

##### Small Intestinal Phase

The gastric pH was adjusted to 6.0 (1 M NaHCO_3_), followed by the addition of bile extract and pancreatin (both in 100 mM NaHCO_3_) to mimic the intestinal environment. Further pH adjustment to 7.0 with NaHCO_3_ reflected the small intestine’s alkalinity. The reaction vessel was purged with nitrogen and incubated at 37 °C, 95 rpm for 2 h (JULABO SW22) to conclude the simulated intestinal digestion.

Following the simulated gastric and small intestinal phases, the resulting digest (10 mL) was transferred to a 15 mL polypropylene tube, purged with nitrogen for minimal contamination, and kept at −80 °C for a maximum of two weeks until analysis. Subsequently, chlorophyll and lutein concentrations were quantified using high-performance liquid chromatography (HPLC). Total phenolic content was determined through the Folin-Ciocalteu colorimetric assay. The digestive stability (%) of phytochemical compounds throughout the simulated digestion was assessed using the following equation:

$$\mathrm{Digestive}\;\mathrm{stability}\;(\%)=\frac{\mathrm{Amount}\;\mathrm{of}\;\mathrm{phytochemical}\;\mathrm{compounds}\;\mathrm{in}\;\mathrm{digesta}\;\times \;100}{\mathrm{Amount}\;\mathrm{of}\;\mathrm{phytochemical}\;\mathrm{compounds}\;\mathrm{in}\;\mathrm{test}\;\mathrm{sample}}$$

#### 2.5.2. Bioaccessibility

Following the simulated digestion, the resulting 10 mL digest was transferred to a 15 mL polypropylene centrifuge tube. To separate aqueous and residual particulate fractions, the sample was centrifuged at 8000 rpm for 90 min at 4 °C using a Thermo Electron LED GmbH D-37520 centrifuge (Langenselbold, Germany). The supernatant (aqueous fraction) was subsequently filtered through a 0.2 μm PTFE membrane to further clarify the solution. Throughout the procedures, subdued lighting and nitrogen blanketing were employed to minimize the potential oxidation of the analytes. Aliquots of the pre-digested sample, digest, and aqueous fraction were gathered and kept at −80 °C for subsequent analysis [19]. Concentrations of chlorophyll and lutein were quantified using high-performance liquid chromatography (HPLC), while total phenolic content was determined through the Folin-Ciocalteu colorimetric assay. Following the simulated digestion, the bioaccessibility of target phytochemicals (chlorophyll, lutein, and total phenolics) in the test sample was expressed as a percentage based on their concentrations in the filtered aqueous fraction using the following equation:

$$\mathrm{Micellarization}\;(\%)=\frac{\mathrm{Amount}\;\mathrm{of}\;\mathrm{phytochemical}\;\mathrm{compounds}\;\mathrm{in}\;\mathrm{micells}\;\times 100}{\mathrm{Amount}\;\mathrm{of}\;\mathrm{phytochemical}\;\mathrm{compounds}\;\mathrm{in}\;\mathrm{digesta}}$$

#### 2.5.3. Bioavailability

##### Cell Culture

This study employed the human colorectal adenocarcinoma cell line Caco-2 (ATCC HTB-37) between passages 22 and 32. The cells were cultured in T-75 flasks and maintained in complete Dulbecco’s modified Eagle’s medium (DMEM) supplemented with 10% heat-inactivated fetal bovine serum (FBS), 1% non-essential amino acids, 1% L-glutamine, and 1% penicillin–streptomycin. The samples were incubated under controlled conditions at 37 °C with 95% air and 5% CO_2_ in a humidified environment. The fresh medium was replaced every 48 h. The experiments were conducted 21 days post-confluence, a stage exhibiting optimal enterocyte characteristics evidenced by marker enzyme activities (alkaline phosphatase and sucrase) [20].

##### Cellular Uptake of Aqueous Fraction

Cellular uptake of the aqueous fraction was investigated using a modified protocol from [19]. Caco-2 cells (passage 22) were seeded at 5000 cells/cm^2^ in T-25 flasks, maintained in a complete medium (replaced every 48 h), and allowed to reach confluence (11–14 days) to develop fully differentiated brush border membranes [21]. Following confluence, pre-washed monolayers were subjected to controlled uptake experiments. Each monolayer was exposed to 5 mL of test medium containing a 1:3 dilution of the aqueous fraction for pre-determined durations of 2, 4, or 6 h at 37 °C. Finally, the test media were removed, and the monolayers were washed with chilled PBS, then the cells were subsequently analyzed by scraping and kept at −80 °C for preservation. Both the culture medium and harvested cells were subjected to high-performance liquid chromatography (HPLC) analysis. Cellular protein concentrations were quantified using the Bradford assay [22] with bovine serum albumin serving as the reference standard.

Formulas for the calculation of cell uptake were as follows:

$$\mathrm{Cellular}\;\mathrm{uptake}\;(\%)=\frac{\mathrm{Amount}\;\mathrm{of}\;\mathrm{phytochemical}\;\mathrm{in}\;\mathrm{cells}\;\times \;100}{\mathrm{Amount}\;\mathrm{of}\;\mathrm{phytochemical}\;\mathrm{in}\;\mathrm{test}\;\mathrm{medium}}$$

##### Transport of Chlorophyll Derivatives, Lutein, and Total Phenolic by Caco-2 Cells

An adaptation of the Chitchumroonchokchai et al. [23] method was employed to assess the transcellular transport of phytochemical compounds across Caco-2 cells. Cells (passage 22) were seeded on Transwell inserts (0.4 μm pore size and 24 mm diameter) at 3 × 10^5^ cells/well with apical and basolateral volumes of 1.5 mL and 2.5 mL, respectively. Experiments commenced 11–14 days post-confluence, coinciding with peak lipoprotein synthesis and secretion [24]. After washing, the confluent monolayers were exposed to 1.5 mL of 1:3 diluted aqueous fraction in the apical chamber and 2.5 mL of complete medium in the basolateral chamber for 6 h. The medium was then removed, the cell layer washed, and 2.5 mL of supplemented media (taurocholate, oleic acid, glycerol) was added to the apical chamber for 16 h to stimulate chylomicron secretion [25]. Both chambers were emptied, monolayers washed, and cells scraped and stored at −80 °C for analysis. The formulae for calculating cellular transport are as follows.

$$\%\;\mathrm{Cellular}\;\mathrm{transport}=\frac{\mathrm{Amount}\;\mathrm{of}\;\mathrm{phytochemical}\;\mathrm{in}\;\mathrm{basolateral}\;\mathrm{chamber}\;\times \;100}{\mathrm{Amount}\;\mathrm{of}\;\mathrm{phytochemical}\;\mathrm{in}\;\mathrm{the}\;\mathrm{test}\;\mathrm{medium}}$$

##### Digesta, Aqueous Fraction, Media, and Cell Pellet Extraction

Caco-2 cell transport of chlorophyll derivatives and lutein was assessed via modified extraction techniques [19]. Thawed digesta, aqueous fractions, and media were extracted three times with acetone/petroleum ether containing BHT. Combined fractions were dried, redissolved in acetone, and analyzed. Cell pellets were extracted similarly, employing SDS-ethanol and a 1:2 acetone/petroleum ether mix. Combined fractions were analyzed after drying and redissolution. Chlorophyll, lutein, and total phenolics were quantified by HPLC and the Folin-Ciocalteu assay, respectively.

### 2.6. Protein Determination

Protein was quantified using the Bradford assay [22] with BSA as the standard. A 1:5 dilution of the Bradford reagent in DI water was prepared and filtered. A total of 20 μL aliquots of samples/standards were combined with 1 mL of the diluted reagent, incubated for 5 min at room temperature, and then measured for absorbance at 595 nm.

### 2.7. Statistical Analysis

All measurements were replicated three times to ensure reproducibility. Statistical analyses were conducted with SPSS software (version 16.0, SPSS Inc., Armonk, NY, USA). Data are presented as the mean ± standard deviation (SD) to summarize the central tendency and variability of the measurements. Comparisons between group means were performed using independent-sample *t*-tests. A statistically significant difference was defined as a *p*-value of less than 0.05.

## 3. Results

### 3.1. Chemical Approximate Analysis

Table 1 summarizes the chemical composition of dried GBC stem powders from 3- and 6-year-old cacti.

### 3.2. Phytochemical Profile of GBC Extract

#### 3.2.1. Chlorophyll and Lutein Profile of GBC Extract

**Figure 1:**
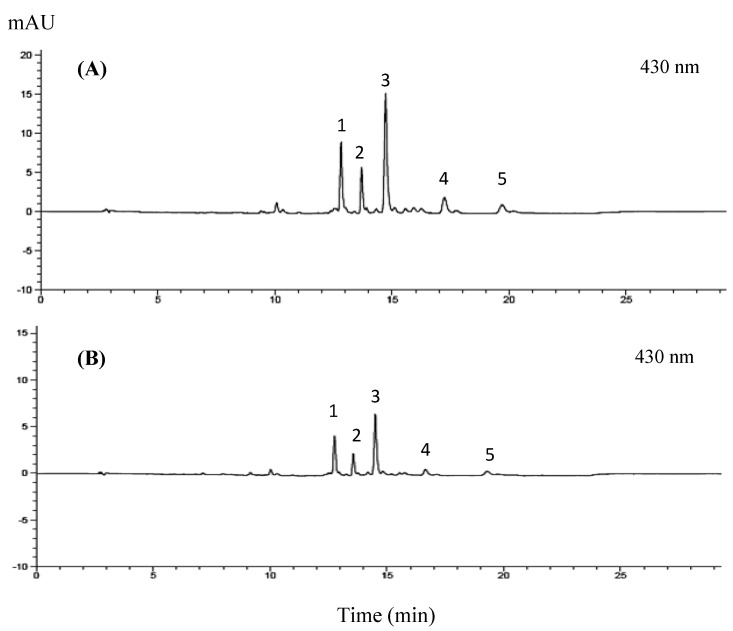
High-performance liquid chromatography (HPLC) analysis revealed distinct profiles of chlorophyll derivatives and lutein in extracts from golden barrel cacti of different ages. Figure shows chromatograms for (**A**) three-year-old cacti, and (**B**) six-year-old cacti. Peaks were identified as follows: (1) lutein, (2) chlorophyll *b*, (3) chlorophyll *a*, (4) pheophytin *b*, and (5) pheophytin *a*.

Chemical analysis of the GBC extract identified lutein and four chlorophyll derivatives: chlorophyll *a*, chlorophyll *b*, pheophytin a, and pheophytin *b* (Figure 1). The detailed composition of the extract is further presented in Table 2.

#### 3.2.2. Total Phenolic Contents

**Figure 2:**
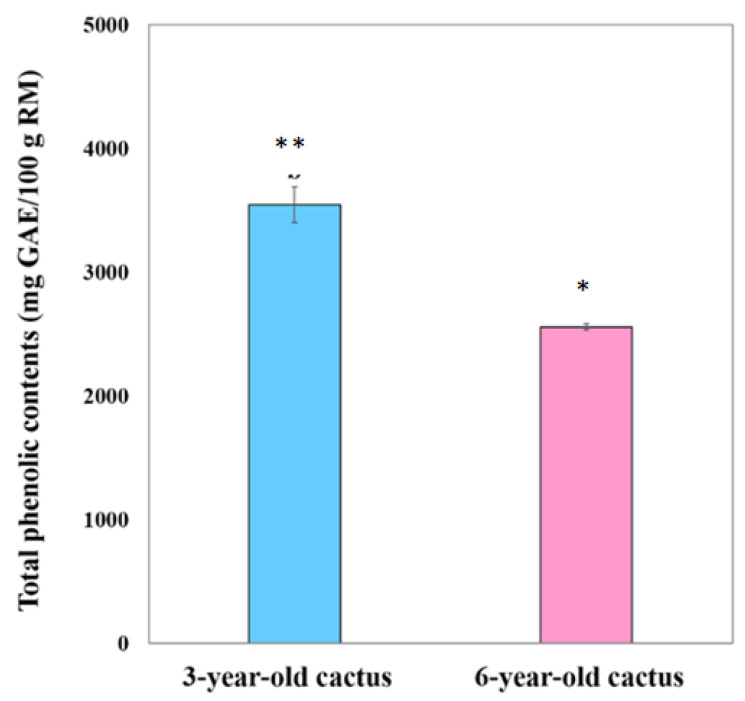
The total phenolic content of extracts from golden barrel cacti of two age groups: 3 and 6 years old. Data points represent mean values accompanied by standard deviations. Statistical significance (*p* < 0.05) of differences between mean values is denoted by different asterisks above the corresponding error bars.

Figure 2 depicts the stark difference in total phenolic content between extracts from 3- and 6-year-old golden barrel cacti. Notably, the 3-year-old extract exhibited significantly higher (*p* < 0.05) total phenolic compounds compared to the 6-year-old counterparts.

### 3.3. Antioxidant Activities

#### 3.3.1. DPPH Free Radical Scavenging Activity

The ability of 3- and 6-year-old GBC extracts to scavenge free radicals (DPPH assay) was assessed, revealing their antioxidant potency through IC_50_ values. As shown in Table 3, the extract from the younger cacti exhibited superior antioxidant activity, as seen by lower IC_50_ values compared to the 6-year-old extract. Additionally, both cactus extracts displayed noteworthy antioxidant capacity when compared to BHT (1 mg/mL), ascorbic acid (1 mg/mL), and established free radical scavengers.

#### 3.3.2. Scavenging Activity of ABTS Radical Cation

The ABTS^+^ radical scavenging activity of 3- and 6-year-old GBC extracts, as measured by IC_50_ values, is presented in Table 4. These values indicate the extract’s antioxidant potency, with lower IC_50_ values signifying greater radical scavenging capacity. Notably, both cactus extracts exhibited comparable or even stronger activity compared to positive controls such as BHT and ascorbic acid.

#### 3.3.3. Ferric Reducing Antioxidant Power Assay (FRAP)

The antioxidant activity of each extract sample, quantified as millimoles of ferrous iron (Fe^2+^) equivalents per gram of raw material, is presented in Table 5. This unit reflects the potential of the extracts to act as a reducing agent, converting ferric iron (Fe^3+^) to ferrous iron (Fe^2+^) and demonstrating its capacity to neutralize free radicals, thereby contributing to its overall antioxidant potency.

### 3.4. Bioaccessibility and Bioavailability

#### 3.4.1. Cytotoxicity

The Caco-2 and HepG2 cell lines served as crucial tools for cytotoxicity assessment in this study. Caco-2, mimicking normal intestinal cells [26] delivered essential preliminary information regarding potential toxicities. The findings informed the selection of suitable concentrations for further cellular uptake and transport studies investigating the behavior of GBC extracts after simulated digestion in vitro.

Cytotoxicity testing at concentrations between 25 and 500 µg/mL revealed minimal toxic effects of GBC extracts from both age groups on Caco-2 and HepG2 cells, regardless of prior in vitro digestion. Consistent with the MTT assay results, LC_50_ values exceeding 200 µg/mL (Table 6) were observed across all conditions, suggesting negligible cytotoxicity compared to control cells cultured in a complete medium. This outcome offers initial evidence for the safety of these extracts and paves the way for further cytotoxicity studies.

#### 3.4.2. Digestive Stability and Percentage of Aqueous Micellar Fraction of Phytochemical Compounds

This study utilized a combined in vitro digestion and Caco-2 cell model to investigate the gastrointestinal fate and cellular uptake of key phytochemicals (lutein, chlorophyll derivatives, and phenolic compounds) present in extracts from 3-year-old and 6-year-old Golden Barrel Cactus (GBC). The quantity of these constituents, categorized as pre-digested, digesta, and separated into aqueous and micellar fractions, is presented in Table 7 for both age groups.

Table 8 offers insights into the bioavailability potential of 3- and 6-year-old GBC extracts by showcasing their digestive stability. Expressed as the percentage of key compounds (lutein, total chlorophylls, and total phenolics) remaining after simulated digestion, this table allows for comparisons between age groups and provides valuable information for understanding the potential absorption and utilization of these bioactive constituents within the human body.

**Figure 3:**
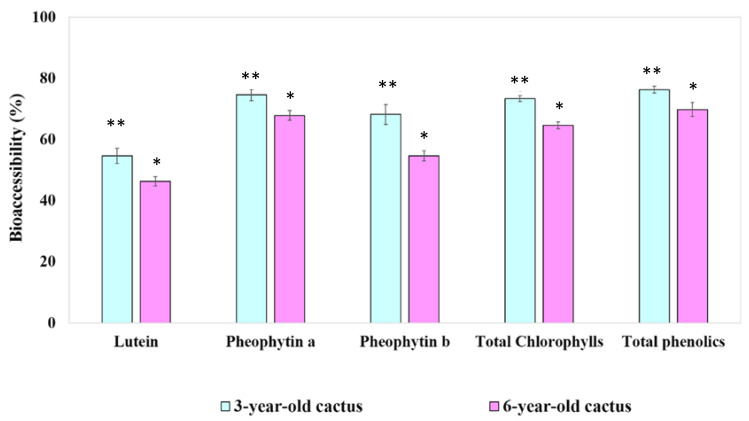
Bioaccessibility of key bioactive compounds in 3- and 6-year-old GBC extracts, namely chlorophyll derivatives, lutein, and total phenolics, following simulated digestion. Data are presented as means ± standard deviations, with statistically significant differences (*p* < 0.05) indicated by distinct asterisks above the error bars.

The ability of key bioactive components in 3- and 6-year-old GBC extracts to overcome digestive barriers and become bioavailable is illustrated in Figure 3. This figure presents the bioaccessibility or micellization efficiency of chlorophyll derivatives, lutein, and total phenolics after simulated digestion, providing crucial insights into their potential absorption and utilization within the human body.

#### 3.4.3. Bioavailability of Phytochemical Compounds

**Figure 4:**
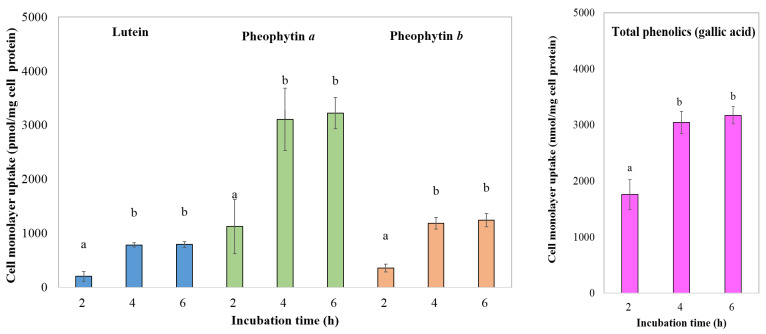
Effect of incubation time on cell monolayer uptake of bioactive compounds from digesta of the 3-year-old GBC extract by Caco-2 human intestinal cells. Data are means ± SD; *n* = 3. Different letters over the error bars denote that the means differed significantly (*p* < 0.05).

**Figure 5:**
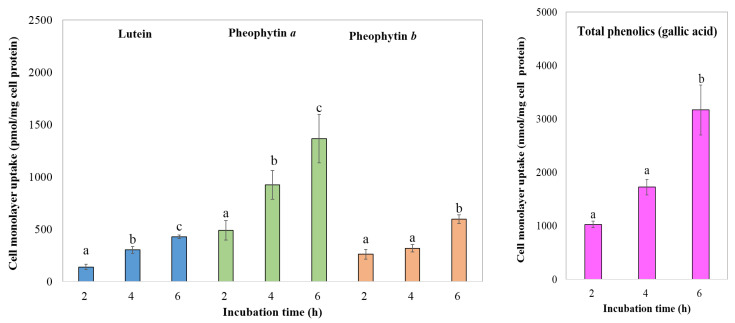
Effect of incubation time on cell monolayer uptake of bioactive compounds from digested of the 6-year-old GBC extract by Caco-2 human intestinal cells. Data are means ± SD; *n* = 3. Different letters over the error bars denote that the means differed significantly (*p* < 0.05).

**Figure 6:**
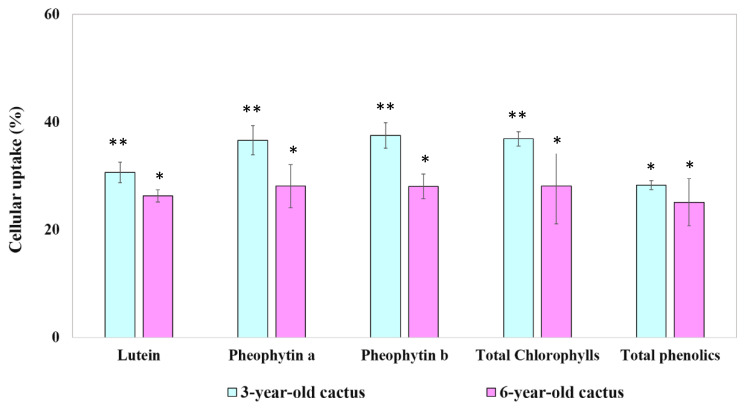
The percentage of cellular uptake of bioactive compounds from digesta of 3- and 6-year-old GBC extracts by Caco-2 human intestinal cells. Data are means ± SD; *n* = 3. Different asterisks over the error bars denote that the means differed significantly (*p* < 0.05).

Understanding the cellular uptake of bioactive constituents in GBC extracts is crucial for evaluating their potential health benefits. Figure 4, Figure 5 and Figure 6 provide insights into the time-dependent uptake of micellarized chlorophyll derivatives, lutein, and phenolic compounds by Caco-2 cell monolayers exposed to aqueous fractions from simulated digestion of extracts obtained from both 3- and 6-year-old cacti. Comparing the uptake profiles across figures and age groups may reveal potential age-related differences in intestinal absorption potential.

#### 3.4.4. Transport of Chlorophyll Derivatives, Lutein, and Total Phenolics by Caco-2 Cells

While the Caco-2 cell line has proven valuable for studying dietary phytochemical transport and metabolism [27], this study aimed to establish a more comprehensive in vitro model replicating the complex process of intestinal absorption. This model incorporates key steps: (1) extraction of phytochemicals from the food matrix, (2) micelle solubilization within the intestinal lumen, (3) cellular uptake by small intestinal epithelial cells, and (4) transport of phytochemicals and metabolites into the lymphatic system. By mimicking these crucial stages, this in vitro model offers increased accuracy for investigating the bioavailability and bioactivity of dietary phytochemicals.

This study evaluated the cellular transport and potential bioavailability of key bioactive compounds in GBC extracts using a Transwell-based in vitro model. Caco-2 cells were exposed to controlled amounts of chlorophyll derivatives (pheophytins *a* and *b*), lutein, and total phenolics from extracts derived from both 3- and 6-year-old cacti. The experiment measured the cellular uptake, packaged into chylomicrons, trans-monolayer transport, and basolateral secretion of these compounds, mimicking the key steps of intestinal absorption [28]. Traditionally, such data are reported as the percentage transfer which represents the efficiency of the cellular transport.

**Figure 7:**
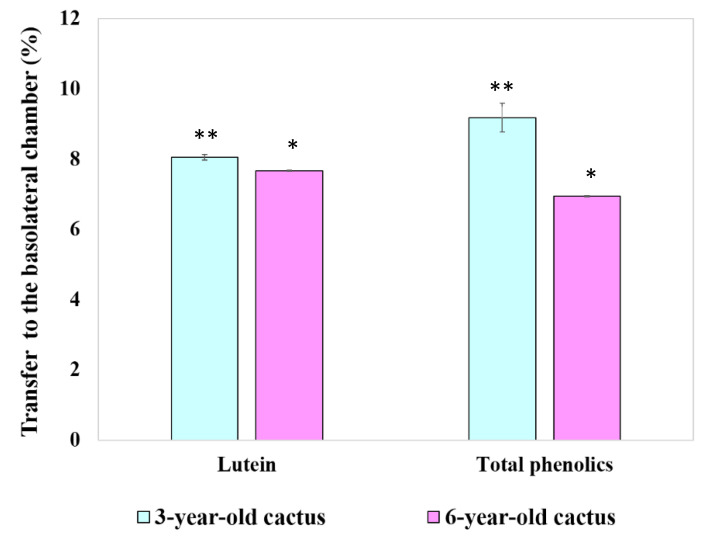
The percentage transport of lutein and total phenolics through differentiated Caco-2 cell monolayers to the basolateral chamber. Data are means ± SD. Different asterisks over the error bars denote that the means differed significantly (*p* < 0.05).

Figure 7 presents the results, which revealed a higher percentage transfer of lutein and phenolic compounds from the 3-year-old cactus extract compared to the 6-year-old counterpart. This suggests that younger cacti may possess enhanced bioavailability of these potentially health-promoting compounds, highlighting the potential age-related variation in absorption efficiency. Further investigations are needed to fully elucidate the underlying mechanisms driving these differences and their broader implications for dietary and functional food applications.

## 4. Discussion

This study revealed significantly higher ash, mineral, and crude fiber content in 6-year-old GBC powder compared to 3-year-old powder. Both age groups, however, exhibited low protein content similar to *Opuntia ficus indica* reported by [29]. Variations in soil, climate [30,31], and potential cactus age [29] might explain these compositional differences. Notably, ash and crude fiber showed a positive association with cactus age, suggesting the potential of older cacti as dietary fiber sources in future supplements. This warrants further research to explore their potential as mineral and fiber-rich dietary additions.

Multiple studies support the trend of higher chlorophyll and lutein content in younger GBC. Holasova et al. [32] observed this decrease in pigment content with age, attributing it to the larger proportion of green tissue in younger cacti. Similarly, Shetty et al. [33] found a negative correlation between age and lutein, suggesting that the higher metabolic activity of younger cacti might drive increased pigment production. Furthermore, Preetham et al. [34] linked the higher antioxidant activity in younger cacti extracts to their potentially greater chlorophyll content, solidifying the connection between age and pigment levels. These findings collectively highlight the influence of age on chlorophyll and lutein content in GBC.

GBC extracts (3 and 6 years old) displayed a significantly higher total phenolic content (3545.35 and 2557.96 mg gallic acid equivalent/100 g dry weight, respectively) compared to both commercial and wild *Opuntia* spp. reported by [35]. This suggests potentially unique, high-value phenolic compounds in GBC. Acetonitrile extraction employed here may have captured diverse phenolic compounds like ferulic and sinapic acids, potentially contributing to the observed abundance. Further investigation into specific phenolic profiles and their bioactivities is warranted. GBC extracts exhibit age-dependent antioxidant activity, with younger plants displaying higher potency. This study confirms previous research, showing significantly higher total phenolic content and stronger antioxidant activity (lower IC_50_ values and ABTS scavenging activity) in 3-year-old extracts compared to 6-year-old ones. However, the FRAP assay results differed from some past studies, suggesting potential influences of extraction methods, analysis techniques, or cactus cultivars. The increased presence of chlorophyll derivatives, lutein, and total phenolics in the 3-year-old GBC extract is directly linked to their enhanced antioxidant potency compared to their 6-year-old counterparts. Further research is needed to clarify these discrepancies and identify the specific bioactive compounds responsible for the observed antioxidant activity.

While the GBC extracts, particularly from younger plants, exhibit promising antioxidant activity, their true health benefits depend on bioavailability, which refers to the ability of the active compounds to be absorbed and utilized by the body. As Walsh et al. [36] outlined, three key factors influence bioavailability: digestive stability, intestinal uptake across epithelial cells, and efficient passage to peripheral tissues. Therefore, future research should delve deeper into these aspects specific to the GBC extracts. This includes investigating if the antioxidant compounds survive digestion, are absorbed adequately, and reach target tissues where they can exert their effects. Studies simulating digestion and intestinal absorption, and potentially utilizing animal models, will be crucial in this endeavor. By bridging the gap between antioxidant activity and bioavailability, we can gain a more holistic understanding of the potential health benefits offered by GBC and its true applications.

Despite similar distributions of lutein, chlorophyll derivatives, and phenolics in 3- and 6-year-old cactus extracts after digestion, the high pheophytin content likely results from the conversion of chlorophyll during the 60 °C drying process, exceeding the 50–60 °C threshold noted by [37] for this transformation. The absence of chlorophyll a and b in the digested and aqueous fractions likely stems from their sensitivity to harsh digestive conditions. Heat and acid, present during the standard gastric phase of in vitro digestion (37 °C, pH 2, 1 h), can degrade these chlorophylls, converting them to Mg_2_+-free derivatives like pheophytins [19,38]. This aligns with observed discoloration from green to brown and agrees with previous findings of complete chlorophyll conversion within 0.5 h at pH 2 [19]. These results highlight the susceptibility of chlorophyll to digestive processes and suggest the need for further investigation into their bioavailability and potential health benefits.

While GBC extracts harbor moderately stable lutein (69.03% and 58.33% digestibility for 3- and 6-year-old cacti, respectively), these findings align with previous research on various plant sources (e.g., spinach) and suggest potential age-dependent declines in stability, like the observed trends in phenolic content and antioxidant activity. However, slight variations in stability values compared to other studies highlight the influence of extraction methods, cactus cultivars, and specific digestion models. Further investigations are crucial to solidify the age-related trend, explore its underlying mechanisms, and identify cultivars with optimal lutein profiles. Additionally, examining interactions between lutein and other cactus components during digestion could shed light on factors affecting its overall bioavailability. Ultimately, this study contributes valuable data to understanding lutein stability in GBC extracts, paving the way for future research to refine our knowledge and maximize its potential health benefits.

While this study reveals significantly higher bioaccessibility of lutein, total chlorophylls, and total phenolics in the 3-year-old GBC extract compared to the 6-year-old one, it echoes existing research on other plants, suggesting a general decline in bioactive compound availability with age. This aligns with observations of reduced β-carotene bioaccessibility in maturing carrots [39], potentially due to changes in cell wall composition or interactions with other plant components. The observed ranking (lutein > chlorophylls > phenolics) resonates with some studies suggesting differential bioaccessibility among phytochemicals based on their structures, interactions with digestive enzymes, or absorption mechanisms. However, limited research on golden barrel cactus necessitates further studies to confirm and refine these findings across cultivars and extraction methods. Additionally, standardizing methodologies would facilitate robust comparisons with other studies. Delving deeper into the mechanisms behind age-related bioaccessibility differences (e.g., cell wall changes, and interactions with other compounds) and exploring the impact of processing methods on bioaccessibility are crucial steps towards optimizing the consumption of golden barrel cactus for maximizing its health potential. This study paves the way for future research to refine our understanding and unlock the full potential of this promising cactus.

The uptake of chlorophyll derivatives, lutein, and phenolic compounds from the 3-year-old golden barrel cactus extract exhibited no significant difference between 4 and 6 h of incubation (*p* > 0.05). This observed plateauing trend aligns with studies on other bioactive compounds, such as β-carotene uptake from carrots reported by Yi et al. [40]. However, there is limited research on golden barrel cactus, necessitating further investigations with standardized methods and broader coverage of cultivars and extraction techniques to confirm and refine these findings.

The cellular uptake of bioactive compounds differed between the cactus age groups. Caco-2 cells appeared to reach saturation after 4 h for extracts from the 3-year-old cacti, indicated by a plateau in the uptake of chlorophyll derivatives, lutein, and phenolics. Conversely, the uptake of these compounds from the 6-year-old cacti continued to significantly increase (*p* < 0.05) between 4 and 6 h, suggesting a later saturation point or potentially a slower overall uptake rate.

There is research indicating that industrial pea preservation techniques (freezing and canning) and subsequent cooking positively influence the bioaccessibility and bioavailability of chlorophyll pigments. Notably, the absence of chlorophyll derivatives in the basolateral chamber following Caco-2 cell uptake warrants further investigation. While potential explanations include degradation during simulated digestion or concentrations falling below detectable levels, as observed with β-carotene [40] and chlorophyll susceptibility [19], definitive conclusions require additional research. Sensitive analytical techniques or concentrated samples could confirm a low-level presence. Utilizing controlled enzyme activities in digestion models or specific inhibitors could isolate the impact of degradation. Additionally, transporter inhibition experiments or investigations into potential efflux mechanisms, inspired by curcumin studies [27], could clarify the role of cellular transport limitations. Delving deeper into these possibilities will refine our understanding of chlorophyll derivative bioavailability from golden barrel cactus extracts and pave the way for maximizing their potential health benefits.

This study reveals higher cellular uptake of lutein and chlorophyll derivatives from a younger (3-year-old) GBC extract compared to older ones, echoing previous research on other plants suggesting age-related declines in bioavailability [39]. This aligns with the idea of age-related changes in plant cell walls or interactions with digestive enzymes affecting compound availability. The observed link between high micellization efficiency and increased cellular uptake resonates with established knowledge of the role of micelles in aiding absorption [37], but limited research on GBC necessitates further studies across cultivars and extraction methods for confirmation. While the lack of significant difference in phenolic compound uptake between age groups warrants further investigation into potential reasons and individual variability, this present study offers valuable insights. Delving deeper into the mechanisms behind age-related micellization changes, understanding specific reasons for differential uptake among compounds, and exploring the impact of processing methods on micellization and uptake are crucial steps toward unlocking the full potential of GBC for human health. This study leads to future research to refine our understanding and maximize the health benefits of this promising cactus.

## 5. Conclusions

This study reveals a significant impact of age on the health potential of golden barrel cacti. Three-year-old cacti boast higher levels and better digestive stability of key antioxidants like lutein and chlorophyll compared to their 6-year-old counterparts. This highlights the exciting potential of younger cacti as a source of bioavailable health-promoting compounds for functional foods and nutraceuticals. Further research is crucial to optimize extraction methods and explore the specific health benefits and ideal dosages for incorporating young golden barrel cacti into this growing field.

## Figures and Tables

**Table 1:** Chemical composition of 3- and 6-year-old GBC powders.

Composition	Content (% Dry Weight)	
	3-Year-Old Cactus Powder	6-Year-Old Cactus Powder
Moisture	0.86 ± 0.06	0.53 ± 0.25
Ash	22.83 ± 0.03 *	25.33 ± 0.02 **
Crude protein	14.48 ± 0.12	14.82 ± 1.33
Crude fat	1.39 ± 0.36	1.02 ± 0.09
Crude fiber	11.18 ± 0.20 *	15.99 ± 0.80 **
Available carbohydrates	49.26 ± 0.54 *	43.64 ± 1.11 **

Note: Data are means ± SD; data in the same row with different asterisks are significantly different (*p* < 0.05).

**Table 2:** Phytochemical content of GBC extract.

Phytochemical Content	GBC Extract	
	3-Year-Old	6-Year-Old
Lutein (µg/g RM)	36.14 ± 0.39 **	30.44 ± 0.45 *
Chlorophyll *a* (µg/g RM)	179.41 ± 2.89 **	115.15 ± 5.83 *
Chlorophyll *b* (µg/g RM)	97.26 ± 0.31 **	91.28 ± 0.80 *
Pheophytin *a* (µg/g RM)	243.46 ± 10.59 **	154.08 ± 5.23 *
Pheophytin *b* (µg/g RM)	6.16 ± 1.45 *	5.87 ± 1.48 *
Total Chlorophylls (µg/g RM)	526.29 ± 10.45 **	366.37 ± 1.22 *

Note: Data are means ± SD; data in the same row with different asterisks are significantly different (*p* < 0.05).

**Table 3:** Antioxidant activities of 3- and 6-year-old GBC extracts by DPPH assay.

Samples	IC_50_ of DPPH Radical Scavenging Activity (mg RM/mL)
3-year-old cactus	112.60 ± 1.42 ^b^
6-year-old cactus	191.90 ± 0.52 ^c^
BHT	0.35 ± 0.04 ^a^
Ascorbic acid	0.08 ± 0.01 ^a^

Note: Data are means ± SD; Data in the same column with different superscripts are significantly different (*p* < 0.05).

**Table 4:** Antioxidant activities of 3- and 6-year-old GBC extracts by ABTS assay.

Samples	IC_50_ of ABTS^+^ Radical Scavenging Activity (mg RM/mL)
3-year-old cactus	44.62 ± 2.43 ^b^
6-year-old cactus	81.84 ± 0.42 ^c^
BHT	0.09 ± 0.003 ^a^
Ascorbic acid	0.04 ± 0.002 ^a^

Note: Data are means ± SD; Data in the same column with different superscripts are significantly different (*p* < 0.05).

**Table 5:** Antioxidant activities of 3- and 6-year-old GBC extracts by FRAP assay.

Samples	FRAP Values (mmol Fe^2+^/g RM)
3-year-old cactus	0.014 ± 0.001 ^a^
6-year-old cactus	0.010 ± 0.002 ^a^
BHT	2.50 ± 0.21 ^b^
Ascorbic acid	8.61 ± 0.14 ^d^

Note: Data are means ± SD; Data in the same column with different superscripts are significantly different (*p* < 0.05).

**Table 6:** Cytotoxic activities of 3- and 6-year-old GBC extracts against Caco-2 cells and HepG2 cells.

Samples	LC_50_ (µg RM/mL)	
	Caco-2 Cells	HepG2 Cells
3-year-old cactus	>200	>200
6-year-old cactus	>200	>200
3-year-old cactus (digesta)	>200	>200
6-year-old cactus (digesta)	>200	>200

**Table 7:** Quantity of phytochemical compounds in pre-digested, digesta, and aqueous micellar fraction from 3- and 6-year GBC extracts.

Phytochemical	3-Year-Old Cactus Extract			6-Year-Old Cactus Extract		
	Pre-Digested	Digesta	Aqueous Micellar Fraction	Pre-Digested	Digesta	Aqueous Micellar Fraction
Lutein (µg/g RM) *	36.14 ± 0.39	24.95 ± 1.19	22.35 ± 0.08	30.44 ± 0.45	17.75 ± 0.17	12.12 ± 0.22
Chlorophyll *a* (µg/g RM) *	179.41 ± 2.89	n.d.	n.d.	115.155.83	n.d.	n.d.
Chlorophyll *b* (µg/g RM) *	97.26 ± 0.31	n.d.	n.d.	91.28 ± 0.80	n.d.	n.d.
Pheophytin *a* (µg/g RM) *	243.46 ± 10.59	159.50 ±2.04	118.97 ± 1.97	154.08 ± 5.23	92.17 ± 7.32	62.59 ± 4.91
Pheophytin *b* (µg/g RM) *	6.16 ± 1.45	38.54 ± 1.04	26.34 ± 1.83	5.87 ± 1.48	29.98 ± 1.19	16.40 ± 0.39
Total chlorophylls (µg/g RM)	526.29 ± 10.45	198.04 ± 2.76	145.31 ± 1.12	366.37 ± 1.22	122.15 ± 6.18	78.98 ± 4.67
Total phenolics (mg GAE/g of RM) **	35.45 ± 1.43	21.43 ± 0.45	16.37 ± 0.19	25.58 ± 0.26	14.55 ± 0.16	10.17 ± 0.34

Note: Data are means ± SD; n.d. = not detected, * Determined by HPLC, ** determined by Folin-Ciocalteu colorimetric assay.

**Table 8:** Digestive stability of phytochemical compounds from 3- and 6-year-old GBC extracts after simulated digestion.

Phytochemical	% Digestive Stability or Recovery	
	3-Year-Old Cactus	6-Year-Old Cactus
Lutein	69.03 ± 2.65 **	58.33 ± 1.38 *
Pheophytin *a*	65.62 ± 3.63 *	59.83 ± 4.70 *
Pheophytin *b*	655.24 ± 187.59 *	534.84 ± 146.47 *
Total chlorophylls	37.64 ± 1.09 **	33.34 ± 1.73 *
Total phenolics	60.52 ± 2.73 *	56.89 ± 1.20 *

Note: Data are means ± SD; data in the same row with different asterisks are significantly different (*p* < 0.05).

## Data Availability

The original contributions presented in the study are included in the article, further inquiries can be directed to the corresponding authors.

## References

[1] Zuniga-Vega M.F., Vazquez-Flores B., Lopez-Cervantes M.G., Perez-Torres A., Guzman-Cruz L.A. (2023). Chemical composition and biological activities of golden barrel cactus (*Echinocactus grusonii*): A review. Molecules.

[2] Gonzalez-Flores M.L., Rodriguez-Garcia R., Reyes-Moreno C., Vargas-Torres A., Guzman-Cruz L.A. (2015). Evaluation of the antioxidant activity and phenolic compounds of extracts from three species of Cactaceae using different extraction methods. Ind. Crops Prod..

[3] Perez-Garcia Y.Y., Hernandez-Muñoz P., Garcia-Mellado G., Moreno-Rojas J.M. (2020). Antioxidant activity and bioactive compounds of eight prickly pear cactus (*Opuntia* spp.) cultivars. J. Sci. Food Agric..

[4] Avila-Flores Y.M., Rodriguez-Ramirez M.I., Rodriguez-Garcia R., Diaz-Sanchez R., Zazueta-Sandoval Y.G. (2022). In vitro digestibility and Caco-2 cell uptake of bioactive compounds from golden barrel cactus (*Echinocactus grusonii*) extracts. Plant Foods Hum. Nutr..

[5] De Leo M., Durante M., Raimondi S., Lombardi V., Borrelli R., De Feo V. (2010). Antioxidant properties and phytochemical profile of *Opuntia ficus-indica* fruit extracts. J. Agric. Food Chem..

[6] Galati E.M., Miceli N., Miceli A., Rozza L., Chisari M.V. (2003). *Opuntia ficus-indica* cladodes can protect gastric mucosa from ethanol-induced lesions in rats. J. Pharm. Pharmacol..

[7] Hernández-Urbiola A., Castañeda-Sauza C., Torres-Tinoco R., Rodríguez-Ramírez M.I., Martínez-Flores I.E. (2012). Nutritional composition and antioxidant capacity of nopal (*Opuntia ficus-indica*, cv. Redonda) at different maturity stages. J. Sci. Food Agric..

[8] Chen H., Wang M., Zhao M., Wang W., He X. (2019). Bioaccessibility and antioxidant activity of phenolic compounds in prickly pear leaves extracted by pressurized solvent extraction. Food Nutr. Res..

[9] Gil-Izquierdo A., Gil M.I., Ferreres F., Tomas-Barberan M.A. (2002). In vitro bioaccessibility and intestinal uptake of beta-carotene from carrot assessed by an in vitro digestion model. J. Agric. Food Chem..

[10] Mineta T., Okubo S., Arai A., Okigami A. (2020). Improvement of chlorophyll uptake from spinach by β-cyclodextrin and its evaluation by Caco-2 cell model. Int. J. Mol. Sci..

[11] Sáez-Plaza P., Perez-Esteban R., Garcia-Alonso S., Moreno-Rojas J.M. (2011). Bioaccessibility and intestinal uptake of phenolic compounds from four prickly pear cactus cultivars using an in vitro digestion model. J. Agric. Food Chem..

[12] Vermaak I., Hamman J., Viljoen A. (2010). High performance thin layer chromatography as a method to authenticate *Hoodia gordonii* raw material and products. S. Afr. J. Bot..

[13] Oonsivilai R., Cheng C., Bomser J., Ferruzzi M.G., Ningsanond S. (2007). Phytochemical profiling and phase II enzyme-inducing properties of *Thunbergia laurifolia* Lindl. (RC) extracts. J. Ethnopharmacol..

[14] Oonsivilai R., Ferruzzi M., Ningsanond S. (2008). Antioxidant activity and cytotoxicity of Rang Chuet (*Thunbergia laurifolia* Lindl.) extracts. Asian J. Food Agro-Ind..

[15] Ksouri R., Falleh H., Megdiche W., Trabelsi N., Mhamdi B., Chaieb K., Bakrouf A., Magné C., Abdelly C. (2009). Antioxidant and antimicrobial activities of the edible medicinal halophyte *Tamarix gallica* L. and related polyphenolic constituents. Food Chem. Toxicol..

[16] Benzie I.F., Strain J. (1996). The ferric reducing ability of plasma (FRAP) as a measure of “antioxidant power”: The FRAP assay. Anal. Biochem..

[17] Wong C.-C., Li H.-B., Cheng K.-W., Chen F. (2006). A systematic survey of antioxidant activity of 30 Chinese medicinal plants using the ferric reducing antioxidant power assay. Food Chem..

[18] Garrett D.A., Failla M.L., Sarama R.J. (1999). Development of an in vitro digestion method to assess carotenoid bioavailability from meals. J. Agric. Food Chem..

[19] Ferruzzi M.G., Failla M.L., Schwartz S.J. (2001). Assessment of degradation and intestinal cell uptake of carotenoids and chlorophyll derivatives from spinach puree using an in vitro digestion and Caco-2 human cell model. J. Agric. Food Chem..

[20] Ellwood K.C., Chatzidakis C., Failla M.L. (1993). Fructose utilization by the human intestinal epithelial cell line, Caco-2. Exp. Biol. Med..

[21] Pinto M., Robine-Leon S., Appay M.D., Kedinger M., Triadou N., Dussaulx E., Lacroix B., Simon-Assmann P., Haffen K., Fogh J. (1983). Enterocyte-like differentiation and polarization of the human colon carcinoma cell line Caco-2 in culture. Biol. Cell.

[22] Bradford M.M. (1976). A rapid and sensitive method for the quantitation of microgram quantities of protein utilizing the principle of protein-dye binding. Anal. Biochem..

[23] Chitchumroonchokchai C., Schwartz S.J., Failla M.L. (2004). Assessment of lutein bioavailability from meals and a supplement using simulated digestion and Caco-2 human intestinal cells. J. Nutr..

[24] Mehran M., Levy E., Bendayan M., Seidman E. (1997). Lipid, apolipoprotein, and lipoprotein synthesis and secretion during cellular differentiation in Caco-2 cells. In Vitro Cell. Dev. Biol. Anim..

[25] O’Sullivan L., Ryan L., O’Brien N. (2007). Comparison of the uptake and secretion of carotene and xanthophyll carotenoids by Caco-2 intestinal cells. Br. J. Nutr..

[26] Okonogi S., Duangrat C., Anuchpreeda S., Tachakittirungrod S., Chowwanapoonpohn S. (2007). Comparison of antioxidant capacities and cytotoxicities of certain fruit peels. Food Chem..

[27] Liu C.-S., Glahn R.P., Liu R.H. (2004). Assessment of carotenoid bioavailability of whole foods using a Caco-2 cell culture model coupled with an in vitro digestion. J. Agric. Food Chem..

[28] During A., Hussain M.M., Morel D.W., Harrison E.H. (2002). Carotenoid uptake and secretion by CaCo-2 cells β-carotene isomer selectivity and carotenoid interactions. J. Lipid Res..

[29] Hernández-Urbiola M.I., Contreras-Padilla M., Pérez-Torrero E., Hernández-Quevedo G., Rojas-Molina J., Cortes M., Rodríguez-García M. (2010). Study of nutritional composition of nopal (*Opuntia ficus indica* cv. Redonda) at different maturity stages. Nutr. J..

[30] Rodríguez-Roque M.J., Rojas-Graü M.A., Elez-Martínez P., Martín-Belloso O. (2013). Soymilk phenolic compounds, isoflavones and antioxidant activity as affected by in vitro gastrointestinal digestion. Food Chem..

[31] Stintzing F.C., Carle R. (2005). Cactus stems (*Opuntia* spp.): A review on their chemistry, technology, and uses. Mol. Nutr. Food Res..

[32] Holasova K., Remsey S., Turova B., Kokoska L., Busta M. (2009). Content of photosynthetic pigments and carotenoids in green and etiolated tissues of cactus *Pereskia aculeata* Miller. J. Sci. Food Agric..

[33] Shetty S., Prakash J., Shantaram S. (2012). Age-dependent variations in carotenoid content and antioxidant activity of cactus pear (*Opuntia ficus indica*) fruit. Food Chem. Toxicol..

[34] Preetham T., Deviprasad J., Raju V.S., Krishnamoorthy R. (2018). Effect of age on antioxidant and antimicrobial activity of *Opuntia ficus indica* Mill. fruit extracts. J. Food Sci. Technol..

[35] Guevara-Figueroa T., Jiménez-Islas H., Reyes-Escogido M.L., Mortensen A.G., Laursen B.B., Lin L.-W., De León-Rodríguez A., Fomsgaard I.S., de la Rosa A.P.B. (2010). Proximate composition, phenolic acids, and flavonoids characterization of commercial and wild nopal (*Opuntia* spp.). J. Food Compos. Anal..

[36] Walsh R.B., Zhang Y., Vodovotz Y., Schwartz S.J., Failla M.L. (2003). Bioavailability of apigenin from single and multiple doses of green tea in humans. Am. J. Clin. Nutr..

[37] Scheer H. (1991). The interfacial factor in the heat-induced conversion of chlorophyll to pheophytin in green leaves. J. Sci. Agric..

[38] Ferruzzi M.G., Blakeslee J. (2007). Digestion, absorption, and cancer preventative activity of dietary chlorophyll derivatives. Nutr. Res..

[39] Perez-Sacalu L., Garcia-Alonso F.J., Granado F., Gil-Izquierdo A. (2020). In vitro bioaccessibility of carotenoids from raw and cooked spinach cultivars using different digestion models. J. Food Compos. Anal..

[40] Yi J., Sun M., Li B. (2011). In vitro digestion and bioaccessibility of carotenoids from orange vegetables. J. Food Compos. Anal..

